# Staff care approaches are associated with mealtime behaviors in older adults with dementia

**DOI:** 10.1093/geroni/igaf122.2925

**Published:** 2025-12-31

**Authors:** Kyuri Lee, Wen Liu

**Affiliations:** The University of Iowa, Iowa City, Iowa, United States; Penn State University, University Park, Pennsylvania, United States

## Abstract

Staff mealtime caregiving is essential to support nursing home residents with dementia, who often experience declines in eating performance and challenging behaviors that interfere with their engagement and food intake during mealtimes. This study examined the associations between staff care approaches and resident behaviors during mealtimes. This study is a secondary analysis of observational data from the OPTIMAL study that tested the impact of a person-centered mealtime care intervention in nursing home dementia care. The sample included 241 full-meal videos, capturing 17 residents with dementia (age 73.9±7.9 years; 88.2% male; 88.2% white, 41.2% married) and 54 direct care staff (age mean ± SD = 44.5±11.6 years; 93.3% female; 80.0% white; 15.8±12.3 years worked as a caregiver). Videos were coded for staff person-centered and task-centered care approaches and resident mealtime behaviors using the refined Cue Utilization and Engagement in Dementia (CUED) mealtime video coding scheme. Resident positive mealtime behaviors were associated with staff person-centered verbal care (β = 0.18, p<.001) and modification of the dining environment (β = 0.41, p=.045), controlling for resident demographic characteristics. Resident challenging mealtime behaviors were associated with staff person-centered verbal care (β = 0.12, p=.019), task-centered nonverbal care (β = 0.98, p<.001), and modification of the dining environment (β = 0.85, p=.004). Staff person-centered care may help promote resident positive behaviors. Minimizing task-centered care may improve resident mealtime challenging behaviors. The association between challenging behaviors, person-centered care, and environmental modifications suggests these strategies may often be reactive rather than proactive. Findings highlight the need for anticipatory care strategies to better support residents who experience mealtime challenges.

